# Hydrogen-Deuterium Exchange Mass Spectrometry: A Novel Structural Biology Approach to Structure, Dynamics and Interactions of Proteins and Their Complexes

**DOI:** 10.3390/life10110286

**Published:** 2020-11-15

**Authors:** Oliver Ozohanics, Attila Ambrus

**Affiliations:** Department of Biochemistry, Institute of Biochemistry and Molecular Biology, Semmelweis University, 37–47 Tuzolto Street, 1094 Budapest, Hungary

**Keywords:** hydrogen/deuterium exchange, mass spectrometry, HDX-MS, protein dynamics, protein conformation, protein complexes, protein-protein interactions

## Abstract

Hydrogen/Deuterium eXchange Mass Spectrometry (HDX-MS) is a rapidly evolving technique for analyzing structural features and dynamic properties of proteins. It may stand alone or serve as a complementary method to cryo-electron-microscopy (EM) or other structural biology approaches. HDX-MS is capable of providing information on individual proteins as well as large protein complexes. Owing to recent methodological advancements and improving availability of instrumentation, HDX-MS is becoming a routine technique for some applications. When dealing with samples of low to medium complexity and sizes of less than 150 kDa, conformation and ligand interaction analyses by HDX-MS are already almost routine applications. This is also well supported by the rapid evolution of the computational (software) background that facilitates the analysis of the obtained experimental data. HDX-MS can cope at times with analytes that are difficult to tackle by any other approach. Large complexes like viral capsids as well as disordered proteins can also be analyzed by this method. HDX-MS has recently become an established tool in the drug discovery process and biopharmaceutical development, as it is now also capable of dissecting post-translational modifications and membrane proteins. This mini review provides the reader with an introduction to the technique and a brief overview of the most common applications. Furthermore, the most challenging likely applications, the analyses of glycosylated and membrane proteins, are also highlighted.

## 1. Introduction

The origins of hydrogen-deuterium exchange date back to the middle of the last century [1,2], when the technique was first applied in the field of NMR spectroscopy to study molecular dynamics. Mass spectrometry came later to this field, in the 1990s, when it became feasible to study large proteins and their complexes. Hydrogen-Deuterium eXchange Mass Spectrometry (HDX-MS) is now a powerful technique for structural protein science, providing detailed insights into protein structure as well as conformational dynamics and function [3]. This approach has already become a frequently applied and well-established methodology in the field of protein structure. Last year, a group of eminent researchers in the field formulated clear recommendations for performing and interpreting HDX-MS experiments [4] and hence paved the way towards truly standardized experiments. The applications of HDX-MS are very versatile: they include studies of protein complexes [5,6,7,8,9,10], ligand binding [11,12,13,14,15,16,17,18,19,20,21,22,23,24,25], dynamic properties like conformational changes and folding/unfolding/refolding [26], conformational changes that arise from allosteric effects [27,28,29], structure and stability of biopharmaceuticals [17,30,31] and epitopes [15,32,33], etc. Unlike several other biophysical techniques, HDX-MS possesses the advantages of no or very high size limit [8] and is useful for studying individual proteins as well as large complexes [34,35]. This is mainly due to the application of proteolysis [14] and radical-induced fragmentation (electron transfer and electron capture dissociation respectively; ETD and ECD) in the course of analysis [36,37,38]. HDX-MS is en route to becoming a routine analytical technique in life science research and drug discovery [26]. With the apparent increase in the number of biopharmaceutical products worldwide, there is more incentive for the use of HDX-MS. As the demand for such measurements has recently been continuously augmenting, an increasingly better commercial availability is also apparent today. The popularity of the technique is also reflected in the growing number of relevant applications reported every year.

While HDX-MS is indeed a very versatile technique, the great number of different applications and their respective reporting styles make the comprehension of this methodology considerably challenging. Moreover, interpretation of the mechanisms that give rise to the measured experimental HDX-MS data are rarely straightforward, due mainly to mass spectrometry signal resolution issues and at times limited spatial resolution. Therefore, it is not enough to have a clear understanding of the experimental design applied; software-aided data interpretation is also necessary.

This review is a short overview of the HDX-MS method, which also focuses on the strengths and weaknesses of the technique as well as data interpretation.

## 2. Theory of HDX-MS

**Figure 1:**
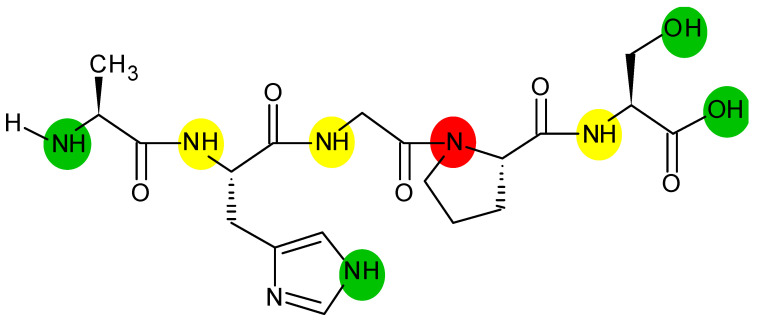
Exchangeable protons in the protein chain. Sites in the protein backbone potentially capable of undergoing H/D exchange are designated with yellow, while additional sites also bearing exchangeable (heteroatom-bound) hydrogens are labeled with green color. Proline possesses no exchangeable hydrogens and thus its backbone amide nitrogen is denoted in red.

The basic principles of HDX-MS have already been reported in several excellent previous articles [20,39,40,41,42], thus here we only discuss it very briefly. In most HDX experiments, deuterons (2H^+^ or D^+^; cation of deuterium) exchange with protons (1H^+^; cation of protium) (Figure 1) in a time-dependent fashion following dilution of the protein into D_2_O, which is buffered usually to a neutral or basic pH [43]; adjustment of the pH secures faster exchange rates relative to those observed under acidic conditions [44].

When preparing the D_2_O buffer, the isotope effect in the course of pH measurement ought also to be taken into account. For a standard glass electrode, this can be carried out simply by adding 0.4 pH units to the readout (at 25 °C) [45]. The experimentally observed rates of the HDX phenomenon are generally in such a range that correlations between the respective exchange rates and conformation/topology of the protein side chains can also be deduced. The actual magnitude of the HDX effect observed is a function of several factors, such as pH, ionic strength, side chain composition, adjacent residues, and interactions with other molecules. Since protein structures are generally rather dynamic entities, most of the amide hydrogens eventually undergo this exchange process as a result of the conformational movements in the protein; nevertheless, there will always be selected sites that are going to exchange extremely sluggishly (only after years). In HDX-MS, backbone amide hydrogens are primarily monitored since their exchange rates can be described on a seconds-to-days timescale, which may be further increased upon structural hindrance, acidic pH, and low temperatures [46].

The H-to-D exchange rate exhibits its minimum at pH 2.54 [44]. The temperature effect was also studied earlier by several groups [2,47,48]. It was shown that temperature dependence is according to the Arrhenius equation (see Equation (1)), which means that changing the temperature from 25 to 0 °C lowers the exchange rate by a factor of 14. In later years, sub 0 °C chromatography was introduced by different laboratories, including the Brock and Engen groups [49,50], as a solution to decelerate the back-exchange process and permit the study of fast-exchanging sites. Recently, Hudgens et al. reported their detailed design for a HDX-MS liquid chromatography (LC) system with two protease columns and two temperature zones (0 and −30 °C) [51]. Factors that also affect the exchange rate are the chemical nature of the solvent and pressure; however, these elements in the protocols are generally already prescribed by the actual experimental setups and the type of proteolysis, among other factors, applied.

(1) $$k_{ch}=k_{ref293}\;*\;\exp\left(-\frac{E_{a}}{R}\left[\frac{1}{T}-\frac{1}{293}\right]\right)$$

Equation (1). Temperature dependence of the H/D exchange rate. k_ref293_ is the exchange rate at 293 K (~20 °C).

### 2.1. Workflow and Equipment

**Figure 2:**
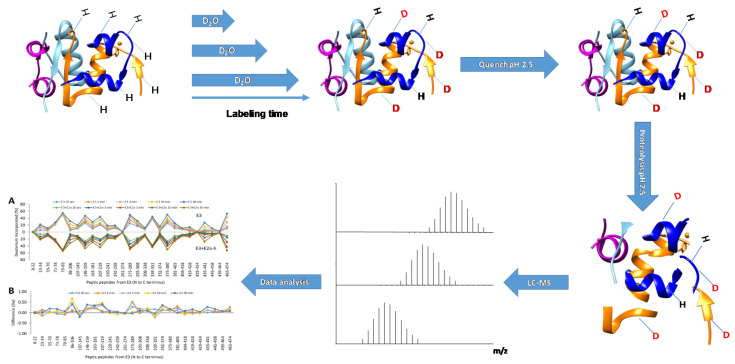
Typical workflow for continuous labeling coupled to “bottom-up” Hydrogen Deuterium eXchange Mass Spectrometry (HDX-MS). In a general continuous labeling workflow, labeling is started by diluting the sample into a D_2_O buffer; after labeling for different time lengths, the reaction is quenched by reducing the pH to 2.5 and the sample is submitted to proteolysis. The sample is analyzed by LC-MS and the resulting raw data is subjected to software aided feature extraction and interpretation.

HDX-MS experiments follow a typical workflow that usually comprises the following major steps: sample preparation, HDX labeling, and quenching; in most protocols a digestion step is also included followed by an LC separation and MS detection. After all these steps, careful and thorough analysis of the experimental raw data is performed. To better deduce the crucial structural and dynamic pieces of information, various methods are generally applied in parallel for interpretation and data representation, with each approach possessing its own strength. A typical workflow is represented in Figure 2.

The sample preparation step is generally unique in every protocol due to the nature and complexity of the actual sample and the usually quite diverging overall experimental design [52]. In pulsed labeling, the labeling time is constant, while the samples also undergo a perturbation/equilibration step for various lengths of time. In contrast, in continuous labeling an identical equilibration step and a labeling step of variable length are performed. In general, the protein of interest is dissolved or diluted in a buffer [53] containing 90–98% D_2_O and salts to secure a suitable solution environment for the protein. The protein sample can originally be in either solution or lyophilized/solid state, although selected proteins are unstable in either state [54]. Since the protein is incubated for a number of different time lengths, multiple conformational states might be sampled in the course of the experiment (various parts of the protein may fluctuate with distinctive velocities).

**Figure 3:**
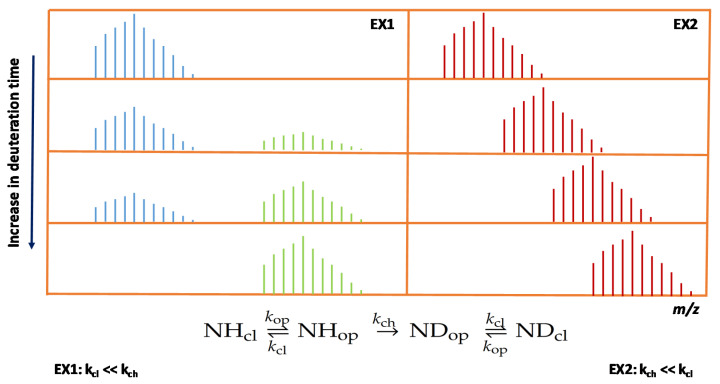
EX1 and EX2 kinetics as observed in the time series of an HDX-MS experiment. In the EX1 regime, two isotopic envelopes were observed and the lower mass envelope disappeared at 100% deuteration.

The protein chain may also exist in two extreme folding states [23,55]. The conformation for which the backbone is solvent accessible and the Gibbs free energy is favorable for hydrogen/deuterium exchange is usually referred to as the “open state”, whereas the so-called “closed state” is much less solvent accessible and the exchange process is generally greatly hindered. Throughout the backbone, where the above phenomena may considerably affect the observed H/D exchange rates, the rate constants of the H/D exchange might be higher or lower than those of the conformation changes, giving rise to two apparent mechanisms, referred to as EX2 and EX1, respectively. When the rate of transition to the open state (k_op_) is much greater than the one of the reverse process (k_cl_, cl: closed state), the protein generally resides in the open state, while the exchange kinetics are predominantly determined by k_ch_ and described by a single envelope of isotopes in the MS signal (EX2 kinetics). In case the protein is principally in the stable closed conformation, the observed kinetics are primarily determined by the relation between k_ch_ and k_cl_. When k_cl_ >> k_ch_, the observed signal is again a single envelope and it is independent of time; the kinetic picture is referred to as EX2. Alternatively, when k_cl_ << k_ch_, the EX1 kinetics give rise to multiple isotopic envelopes in the MS spectra, whose intensity ratios are dependent on the H/D exchange times. An example of these processes is shown in Figure 3. In case of native proteins, the EX2 kinetics are much more common [56,57], but the EX1 kinetics are also observed at times [10,25,55,58,59]. Occasionally, the relevant H/D exchange rate and the respective rate of the studied conformational change are of similar magnitudes; this results in a mixed-type kinetics. Here, the shift from the lower masses is much slower and two distinct envelopes are observed even after prolonged exchange times [58].

The quenching step in the workflow is performed in order to greatly decelerate the deuteration process in the protein via reducing the temperature and pH; the target pH and temperature are ~2.5 and <0 °C, respectively. The quenching buffer usually also contains a denaturing agent (like guanidine hydrochloride) and a reducing agent (e.g., tris(2-carboxyethyl)phosphine (TCEP)) to break the disulfide bonds [60]. Reducing agents often work sub-optimally under the conditions of HDX experiments; online electrochemical reduction of disulfides has proved to be a better solution in various applications [61,62]. (This method is now commercially available).

After quenching, online digestion, with pepsin or selected other enzymes (see below), is carried out; the optimal operation of the digestive enzyme under cold and acidic conditions is necessary. The choice of enzyme applied is largely dependent on the actual instrumentation and the degree of automation. In most research groups, either commercial (fully automated) or in-house-constructed systems are used, where the enzyme is ligated onto a chromatographic matrix; such pre-packed columns are now commercially available. For home-packed columns, Protease XIII and *Aspergillus niger* Prolyl Endo-protease are also frequently used [63,64]. Recently, in the James A. Carroll laboratory, the concept of a dual protease column was introduced and used for the analysis of monoclonal antibodies [65]. The enzyme is usually placed together with the analytical column into a chilled enclosure; however, in selected applications a separate heating unit is used to keep the enzyme at 10–15 °C.

To enable detection of deuteration in case of complex mixtures, an LC-MS workflow is generally preferred, although microchip capillary electrophoresis can also be used [66]. The separation step can be performed on a nanoscale LC, while analytical and microflow systems are also commonly used. The reversed-phase chromatography step is generally carried out at or close to 0 °C, since the back-exchange rate is a function of the applied temperature; in several applications, sub-zero degrees are applied with glycols administered into the buffer against freezing [49,50]. Evolution of the instrumentation commenced with in-house-built enclosures coupled to conventional high performance liquid chromatography (HPLC) systems. HDX-MS robots were later developed by several teams and some replicas of these were even commercialized, for example, by Leap Technologies, Inc. (currently Trajan Scientific and Medical, Inc.). Soon, the very first HDX-UPLC system was commercialized by Waters (originally published by Engen [67]) and research groups also started repurposing pipettor robots for HDX sample preparation [31]. Recent research sponsored by Eli Lilly showed that complete automation is also possible via building a decoupled automation platform [13].

Successful analysis demands on high resolving power and sensitivity from the MS instrumentation; ion mobility separation is an increasingly popular addition, as it greatly enhances the performance of the system [68]. Even in this case, purification of the target protein to higher homogeneity leads to better quality results with less signal interpretation errors. In terms of adequate mass spectrometers for HDX-MS, Orbitrap and quadrupole time-of-flight (QTOF) analyzers coupled with an electrospray ion source are generally utilized. This setup is usually able to secure the demanded resolution and sensitivity and is suitable for online coupling to liquid chromatography. Alternatively, one may use a matrix-assisted laser desorption/ionization (MALDI) ion source and QTOF analyzer, since this approach will not produce hydrogen/deuterium scrambling; nevertheless, this setup has the disadvantages of more cumbersome operation and the demand of specific instrumentation for off-line separation and spotting [69,70,71].

Data analysis and visualization of the results are active areas of research. There are several published protocols to guide the analysis of HDX-MS data; however, each solution possesses advantages and disadvantages. Nevertheless, manual validation of the results is still a sensible approach. Available software tools include Mass Spec Studio [6], HDX Workbench [72], DECA [73], Hydra [74], ExMS [75], HDX-Analyzer [76], HexIcon 2 [77] and HDX-Viewer [78], along with commercial solutions from mass spectrometry vendors. A more detailed overview of these tools can be found in a computation-focused review by Claesen [79]. Data acquisition is relatively straightforward for HDX-MS, unlike interpretation of the results. Depending on the type of experiment, the resultant peptides are to be identified and correction for back-exchange (if any) ought to be performed.

The minimal effect size that should already be taken into account is also an issue. When using state-of-the-art instrumentation, natural variation in isotopic distribution and typical measurement errors secure a minimum effect size of 0.1–0.5 Da (lower if ECD/ETD is used). According to a paper by Weis, the significance threshold should also always be estimated, preferably by the Welch’s *t*-test [80]. Another issue is the calculation of residue level data. While one might obtain suitable overlapping fragments from pepsin digestion, the deuteration levels observed are not additive measures of true deuteration. HDX-MS data analysis progressively shifts towards automation; however, manual validation is still required [81]. It is much more challenging to analyze deuteration patterns than to simply report the average number of hydrogens exchanged [82].

The following are the steps in the analysis: generation of a peptide list, identification of target peptides in the LC-MS data, extraction of features (isotopic patterns), calculation of deuteration levels, and representation of the raw data. Sometimes kinetic parameters for the H/D exchange process itself and the conformational changes of the protein can also be deduced from HDX-MS data [1,9,23,25,40,47,58,83,84,85,86]. For peptide identification, a theoretical and an experimental peptide list can be used together. The theoretical list can be generated in silico, e.g., with FindPept on the Expasy website, taking into account the specificity of the applied protease. The experimental list can be obtained from an MS/MS experiment using a non-deuterated sample; the resulting peptide list is to be matched with the target protein sequence. While the actual experimental list better reflects the truly available information, in possession of a high enough mass accuracy and adequately purified sample the theoretical list can also be used for further analysis [6,40,87,88]. In case peptide identification in subsequent measurements uses retention time data, retention time alignment of the LC-MS data is usually also required. Efficacy of peptide identification can be further enhanced by using ECD/ETD fragmentation; these methods can also be applied to deuterated peptides. If the mass spectrometer is properly tuned, as described in the paper by Rand et al. from 2010, there is little to no deuterium scrambling. [5,89,90,91,92]. Extraction of features and calculation of deuteration levels are negatively affected by co-elution and EX1 (or mixed EX1/EX2) kinetics. There are several algorithms which have been developed to cope with such problems [57,58,77,79,88,93]; discussion of these algorithms is beyond the scope of this article. Usually, the centroid of the isotopic envelope is calculated as a measure of the average deuteration level based on which a mass shift will be calculated (usually denoted as ∆D). It is recommended to correct the results for back-exchange by including samples that were subjected to extensive, theoretically 100%, deuteration [4,46]. This is mandatory for projects dealing with long-term comparability. In these cases, the mass shifts should be calculated according to Equation (2) [94]. When system suitability tests are performed, the back-exchange levels ought also to always be monitored. A system works properly when no or only a few peptides display back-exchange levels above 50% [4,94]. The centroid is a good measure in EX2 kinetics, but it is not well suited for any other types of kinetics or data originating from multiple proteins. A solution to this issue was provided by Guttman et al. via introducing estimation type of algorithms for peak distribution (based on a binomial distribution function) and peak widths (by Gaussian functions) [95]. This type of analysis was incorporated into DECA [73] and the latest version of HX-Express [96]. Another approach is implemented in the HDsite software [97], which uses isotopic peak amplitude ratios and iterative fitting of isotopic envelopes, while it also involves back-exchange correction.

(2) $$\Delta Dcorr=\frac{m_{exp}\;-\;m_{0}}{m_{100\%}\;-\;m_{0}}\times N$$

Equation (2) Calculation of the corrected deuterium exchange (∆Dcorr). m_exp_—experimentally determined centroid, m_0_—centroid of the non-deuterated peptide, m_100%_—centroid of the fully deuterated peptide, N—number of exchangeable amide hydrogens in the peptide, calculated from the length of the peptide excluding prolines and the N-terminal residue.

**Figure 4:**
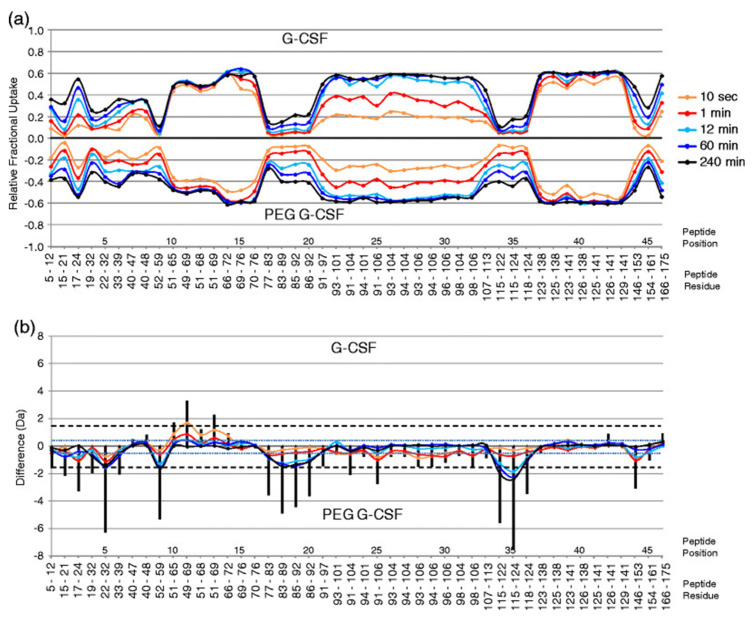
Investigation of the effects of PEGylation on G-CSF. On a representative butterfly chart, forty-six peptides of G-CSF and PEG-G-CSF are compared in terms of (**a**) relative change in deuterium levels and (**b**) absolute mass difference. Relative D-uptake is obtained by dividing the deuterium level (in Da) by the total number of backbone amide hydrogens that could have become deuterated. In (**b**), the blue dotted line is set at 0.5 Da (both positive and negative difference) as the threshold for significant differences. Reprinted with permission from Wei et al. Using hydrogen/deuterium exchange mass spectrometry to study conformational changes in granulocyte colony stimulating factor upon PEGylation. Journal of the American Society for Mass Spectrometry **2012**, *23*(3), 498–504.

Once the derived pieces of information are available, a number of further tasks are to be performed. First, if a 3D structural model is available, one may represent the changes between two states on the structure, which facilitates the understanding of the molecular motions and interactions involved. For instance, HDX-Viewer [78] generates a heatmap of deuteration in the protein. Time series data (alterations in ∆D with time) can be displayed in multiple fashions. The most popular representations are protein-wide stacked column plots and butterfly plots; a representative example of the latter is shown in Figure 4 [98]. On these plots, one may represent the sequence or residue positions in the protein and the absolute mass shift or percentage of the theoretical maximal mass shift for a given sequence region. It is crucial to display both measures since shifts by 0.5 Da or smaller have proved to be unreliable in further analyses [99].

### 2.2. Strengths and Weaknesses—Comparison to NMR

The main strength of HDX-MS is that it can study proteins that are hard to analyze by other means. H/D exchange had traditionally been studied by NMR, but after the MS-based counterpart was devised, high molecular weight proteins (even complexes of MDa size) could be investigated with relative ease. Exchange rates that derive from HDX-NMR measurements provide orthogonal reference measurements to HDX-MS, while using a different methodology. Specific NMR experiments can analyze intact proteins and measure the exchange rate constants of individual amide sites [100]. It is well established that pH affects the H/D exchange rate [101]; this phenomenon can be exploited to shift exchange rates to a range accessible by NMR. The principal sources of error, however, are different for the two methods. Comparison of the results from the two sources can be carried out only where the data overlap. The degree of accord of the results show how reliable the HDX-MS results are for a protein. The Englander research group performed a comparison on a double mutant Staphylococcal nuclease (SNase) using HDX-MS and 2D NMR. The NMR-derived exchange rate constants of the amide hydrogens lay within a range of ±14% [100]. Using HDX-MS, they measured the D uptake by SNase at four different pH values with exchange time intervals between 10 s and 4 weeks. The measured exchange rates varied by more than a factor of 100. They applied the method by Guttman [95], fitted the coefficients for each peptide and obtained the back-exchange-corrected D-occupancy at each amide site. The time-series of D-occupancy at each site was fitted to an exponential decay function, which directly yielded the site-resolved HDX rate coefficients. Comparison of the results revealed that, out of the 99 sites, 81 residues were within a threefold range of the measured NMR protection factors. This study showed the extent to which HDX-MS measurements can be used to calculate kinetic parameters as well as showing the demand for correction of the auxiliary effects inherent in the MS-based method. Although HDX-MS can be used to map the structures of proteins, the amino acid resolution of the technique is, however, highly dependent on the sequence of the target protein and the type of proteolytic digestion applied. This may be further complicated by the potential deuterium/hydrogen scrambling effects in the course of the analysis. If feasible, both top-down (intact protein based) and bottom-up or middle-down (digested protein based) analyses ought to be carried out.

Back-exchange is a major problem in HDX-MS analysis. While precisely controlled experimental conditions may minimize this effect, chromatographic separation is responsible for most of the back-exchange, mainly due to the length of the process. This results in significant loss and alteration of signal, and biased measurement results, as not all peptides behave in the same way and significant differences exist between the exchange rates of peptides with different lengths and/or sequence. Still, HDX-MS is a good choice for studying hydrogen bonding networks and folding in proteins, especially when only a limited amount of sample is at one’s disposal. Applied in conjunction with other methods, like chemical cross-linking, HDX-MS helps elucidate structure and conformational dynamics in individual proteins and large protein complexes [102]. When analyzing protein complexes, binding or interaction sites can also be identified. Additionally, a key strength of HDX-MS is its complementarity with cryo-electron microscopy (cryo-EM) [103]. Cryo-EM and HDX-MS can both provide unique pieces of information that validate one another. Recent methodological advancements in both techniques also now permit the study of large and complex systems. Measuring a protein in both isolation and complex by HDX-MS may reveal how intermolecular interactions affect its dynamics and allosteric regulation. It is a great advantage that cryo-EM may help interpret HDX-MS results, especially when crystallographic information is not available.

## 3. Applications

A number of HDX-MS applications can already be considered as routine [26,39]. Conformational analysis of proteins is now used in the pharmaceutical industry for comparability and stability analyses as well as development of biosimilar products [26,34,56,68,104,105]; regulatory authorities now accept HDX-MS for biosimilar structure confirmation.

There is a plethora of examples for the application of HDX-MS in protein interaction studies [21,106]. More challenging are those applications, where the protein is part of a large protein complex, such as a nuclear pore complex [34,35], embedded in a membrane [15,107], or disordered [108,109]. Selected protein modifications, like glycosylation and disulfide bonding, hinder proteolysis and/or mass analysis [26]. An exciting field of development is ’millisecond pulsed labeling’ that can reveal information about intrinsically disordered proteins and allosteric effects, and has the ability to detect co-existing folding states [66,102,110,111,112,113,114]; for millisecond labeling, microfluidic devices need to be applied [111,112,113,114].

### 3.1. Analysis of Protein Conformation and Folding

Correct conformation of proteins is of utmost importance for their function. For a protein, a folding-unfolding equilibrium is constantly present in solution. Each process can take place on a wide timescale, from microseconds to several seconds, depending on length and sequence [115]. These mechanisms are inherently studied in the course of an HDX experiment; folding equilibria give rise to a statistical ensemble of particles, each with a separate deuteration pattern. The resultant data set is a time-averaged representation of the flexibility and molecular motions of the target protein. Alternative folding may be induced, for instance, upon binding to partners or changes in the amino acid order, as in the case of single nucleotide polymorphisms. Alterations in flexibility/solvent accessibility are more pronounced when the structural changes occur in less structured or completely unstructured regions of the protein. These types of changes are quite cumbersome to study via crystallographic techniques, but are readily assessable with HDX-MS. One of the classic uses of HDX-MS is in fact to compare conformers of proteins. This application is used in, e.g., analysis of protein dynamics [25,54,116], investigation of single nucleotide polymorphisms and their effects in disease states [59,68,117], and exploration of the mechanisms of protein aggregation and amyloid fiber formation [84,86,118]. HDX-MS is used in conjunction with native mass spectrometry and chemical cross-linking to better understand the changes in a protein structure [119,120].

Our laboratory previously analyzed the effects of ten disease-causing amino acid substitutions in the human Dihydrolipoyl dehydrogenase (hLADH, hE3) [59]; hLADH is a common E3 subunit of the mitochondrial alpha-keto acid dehydrogenase complexes. Most of the analyzed mutations lead to severe metabolic decompensations that manifest in cardiological and neurological symptoms [121,122]. As X-ray or NMR structures of these mutants were not available at the time, HDX-MS gave important insights into why these mutants are pathogenic. It was speculated earlier that the observed structural alterations led to dysfunction via affecting the active site, cofactor-binding regions, or protein interaction interfaces. Several of the structural conclusions were in line with those of MD simulations [123]. All these findings were later confirmed in part by X-ray crystallography [124,125].

### 3.2. Analysis of Protein Interactions

Studying interactions with various ligands and other protein partners is an important topic in protein science. Such an investigation might prove to be a difficult task, since some protein complexes have MDa sizes and high complexity, being often made up of several subunits, sometimes even with an unknown stoichiometry. Binding of a ligand in most cases causes changes in the protein structure via either direct/local or allosteric effects. HDX-MS is an adequate technique for detecting structural changes induced by ligand binding as well as localizing allosteric regulatory sites [16,20,126]. Since structural alterations due to direct/local effects and allostery are cumbersome to distinguish, expression of the relevant binding domains as well as complementary methods, such as Förster resonance energy transfer (FRET), chemical cross-linking [10,127,128,129] and mutagenesis [130], should often be also employed. A good example of this principle was reported by Masson et al. in 2016 [131], where the authors studied the protein called Phosphatase and tensin homologue deleted on chromosome 10 (PTEN), an important tumor suppressor. Activity of this enzyme is controlled by phosphorylation at multiple sites. The C-terminus is disordered, which makes its examination quite difficult. A variant of PTEN, PTEN-long (PTEN-L), has an N-terminal extension of 173 residues causing movement of the protein from one cell to another. The study employed both HDX-MS and FRET to show that phosphorylation at either terminus affects the activity of the enzyme. Using HDX-MS, a membrane-binding element was also identified at the N-terminus of PTEN-L.

Alterations in conformational dynamics upon ligand binding were described in several earlier reports [11,12,14,17,19,21,22,23,25,126,130]; in selected studies, an allosteric effect could also be identified. A great example of perturbed conformational dynamics was published by Zhou et al. in 2017 [25], where HDX-MS analysis shed light on the role of conformational dynamics, which was considerably altered upon ligand binding, in the mechanism of action of the DXPS enzyme; the closed conformation of DXPS proved to be critical for stabilization of the transition state, whereas the open conformation is apparently required for releasing the lactyl-thiamin diphosphate final product.

In protein complexes, HDX-MS is capable of revealing the binding interfaces as well as multimerization-induced structural changes in more distant regions; the latter can be considered to be allosteric effects. Studying protein complexes under the size of 150 kDa is considered to be routine work today [26]. The obtained information may be complementary to research data collected by various other methods and could potentially also reveal the relationships among the interactions and functions of the examined proteins. A significant number of previous studies on protein complexes explored antibodies. A recent paper, for instance, analyzed the binding of IgG1 to the neonatal version of the Fc receptor [132]. A more challenging application of HDX-MS is seen in a publication by Lim et al. from 2017 [133], in which the authors studied the assembly of the viral capsid in the Dengue virus (serotype 2). This study demonstrated temperature-dependent dynamic hotspots on the surface of the capsid and concluded that HDX-MS had successfully been applied to determine potential epitopes on the virus. Epitope mapping is the procedure of identifying binding sites that participate in antigen-antibody interactions. Identification of epitopes is critical for developing novel antibody-based therapeutics and vaccines. Revealing epitopes might also contribute to the elucidation of the mechanism of action of the target protein. Strength of HDX-MS in epitope mapping originates in its high sensitivity and great tolerance of protein size. Even large protein complexes, like the histocompatibility complex, can be addressed in terms of epitope mapping [18].

While the previously reviewed applications were on the routine side of HDX-MS, targeting large complexes is certainly not an easy task. Although characterization of multi-protein complexes is one of the strengths of HDX-MS, there are still several issues regarding instrumentation and experimentation. Among others, peak capacity of the applied instrumentation is often limited, while back-exchange increases with time, for example. There are several potential ways to address these issues, e.g., via using orthogonal separation, such as ion mobility [21,68,88,134,135], or reducing back-exchange by applying sub-zero chromatography (see above) [49,50]. Currently, studying large complexes is the focus of research, as most cellular processes make use of an assembly of multiple proteins. A study by Harrison et al. from 2016 [34] indicates that it is already feasible to analyze large signaling complexes by HDX-MS. The authors suggested that HDX-MS data should be correlated with those of functional assays to gain deeper insights into the studied system.

Another great example for a large protein complex is F0F1-ATP synthase. This complex was studied, among others, by Vahidi et al., and using HDX-MS the authors gained intriguing structural insights into three key states in the course of catalysis: the ATP-bound, active catalytic, and uncoupled states [136]. Another important conclusion could also be drawn: uncouplers must act on the γ subunit, since the mechanical stress exerted by this subunit on the shaft disappeared when an uncoupler was present.

### 3.3. Proteins with Glycosylation and Disulfide Bonding

Most of the circulating human proteins are glycosylated. The presence of glycosylation results in higher heterogeneity, as N-glycosylated proteins have multiple variants, present in different amounts. Glycosylation also often hinders digestion and identification of the resulting peptides. However, among the most studied proteins several are antibodies [26,116,132], which contain glycosylation as well as disulfide bridges. To be able to work with these proteins, a solution is to de-glycosylate and reduce them. In 2016, Jensen et al. reported that PNGase A, often used in HDX-MS analysis, is able to work at acidic pH and low temperature [137]. Incorporating this enzyme into the quench buffer along with 0.25 M TCEP appears to be a solution for the analysis of glycosylated (and disulfide-containing) proteins. In case information about the effect of the glycan structure on conformation is desired, a potential resolution is the application of ECD/ETD, which improves MS identification and spatial resolution, while also enabling identification of deuterated glycans (which would otherwise lead to measurement bias) [5,89,90,91,138,139].

### 3.4. Membrane Proteins

HDX-MS of membrane proteins is still a great challenge while these proteins are increasingly important targets in drug discovery. Experimental issues arise not only from the in-solution instability of these proteins, but also from the need to use micelles or liposomes as membrane mimetics [34] and detergents for solubilization. These proteins are also difficult to express, therefore NMR and X-ray studies are rather rare. HDX-MS can be and was used in several published studies to characterize G-protein-coupled receptors (GPCRs), which are involved in cellular signal transduction [140]. These receptors are important targets in drug development; hence any structural insight is valuable. The first study in this field was published by Zhang et al. in 2010, where a detergent was used to solubilize a β2-adrenergic GPCR [24]. There were several experimental challenges to solve, but the authors eventually succeeded in optimizing the detergent amount, composition of the quenching solution and key parameters of the LC step. A major challenge was the presence of large amounts of detergents in the quenched sample. Detergents and lipids negatively impact the performance of the LC-MS system, therefore only limited amounts of these compounds should be present when the sample reaches the analytical column. Trap/pre-columns are used to bind lipids and/or detergents, but these generally have relatively short life spans and require regular and extensive cleaning. Most membrane proteins are highly hydrophobic which necessitates the use of either a C8 or C4 LC column. This feature also affects the sequence coverage map. Undigested membrane proteins are rich in hydrophobic residues and thus tend to aggregate in solution leading to clogging of the analytical system. Recently, Anderson et al. published a method for the automated removal of phospholipids [141]. The authors utilized ZrO_2_ particles to bind and sequester phospholipids. Implementation of the method was performed using a Leap X-Press robot (Trajan Scientific and Medical) and nano-filter vials; however, manual execution is also possible. The described method adds roughly 1 min of extra sample preparation time, while also comprising a TCEP-based disulfide bond reduction step.

## 4. Conclusions

HDX-MS is evolving to become a routine tool in structural biology. Availability of automation and commercial HDX systems make it a good choice for even high-throughput screening demanded in the pharmaceutical industry. The method is uniquely suited to analyze conformational dynamics of proteins in solution for both small and large systems and is complementary to many biophysical methods, such as cryo-EM. The latest developments broaden the range of problems that can be tackled with by HDX-MS and display the inherent possibilities in this methodology. Challenging targets and active development in the field of supporting software push HDX-MS to the frontline of biophysical and structural biology research.
